# Expression, Purification, and Structural Insights for the Human Uric Acid Transporter, GLUT9, Using the *Xenopus laevis* Oocytes System

**DOI:** 10.1371/journal.pone.0108852

**Published:** 2014-10-06

**Authors:** Benjamin Clémençon, Benjamin P. Lüscher, Michael Fine, Marc U. Baumann, Daniel V. Surbek, Olivier Bonny, Matthias A. Hediger

**Affiliations:** 1 Institute of Biochemistry and Molecular Medicine (IBMM), and NCCR TransCure, University of Bern, Bern, Switzerland; 2 Department of Obstetrics and Gynecology, University Hospital of Bern, Bern, Switzerland; 3 Department of Clinical Research, University of Bern, Bern, Switzerland; 4 Department of Pharmacology and Toxicology, University of Lausanne, Lausanne, Switzerland; University of Cambridge, United Kingdom

## Abstract

The urate transporter, GLUT9, is responsible for the basolateral transport of urate in the proximal tubule of human kidneys and in the placenta, playing a central role in uric acid homeostasis. GLUT9 shares the least homology with other members of the glucose transporter family, especially with the glucose transporting members GLUT1-4 and is the only member of the GLUT family to transport urate. The recently published high-resolution structure of XylE, a bacterial D-xylose transporting homologue, yields new insights into the structural foundation of this GLUT family of proteins. While this represents a huge milestone, it is unclear if human GLUT9 can benefit from this advancement through subsequent structural based targeting and mutagenesis. Little progress has been made toward understanding the mechanism of GLUT9 since its discovery in 2000. Before work can begin on resolving the mechanisms of urate transport we must determine methods to express, purify and analyze hGLUT9 using a model system adept in expressing human membrane proteins. Here, we describe the surface expression, purification and isolation of monomeric protein, and functional analysis of recombinant hGLUT9 using the *Xenopus laevis* oocyte system. In addition, we generated a new homology-based high-resolution model of hGLUT9 from the XylE crystal structure and utilized our purified protein to generate a low-resolution single particle reconstruction. Interestingly, we demonstrate that the functional protein extracted from the *Xenopus* system fits well with the homology-based model allowing us to generate the predicted urate-binding pocket and pave a path for subsequent mutagenesis and structure-function studies.

## Introduction

GLUT9 (SLC2A9) membrane transporter is distinct among other members of the glucose transporters (GLUT or SLC2) family due to its substrate specificity and sequence identity. While the majority of 14 members of the GLUT superfamily transports glucose or other monosaccharides [1], GLUT9 was shown to transport essentially urate [2], [3]. Transepithelial urate transport is of critical importance to urate homeostasis in humans, as the lack of the urate catabolizing enzyme uricase elevates the serum uric acid (SUA) levels six to ten times compared to other mammals with functional uricase. High SUA levels increase the risk for uric acid precipitation illustrated by gout flairs, tophi and kidney stone formation, but hyperuricemia, independent of crystal formation, has also been linked with hypertension, atherosclerosis, insulin resistance, and diabetes [4]. Uric acid excretion depends for about one third on intestinal secretion and for about two-thirds on the complex transport (reabsorption and secretion) in the proximal tubule of the kidney. Reabsorption depends mainly on the transport activity of the apical SLC22A11 (URAT1) and the basolateral SLC2A9 (GLUT9), while secretion is driven by the ATP-dependent ABCG2. Loss of function mutations in both SLC22A11 and SLC2A9 conduct to familial hypouricemia, an autosomal recessive trait characterized by hypouricemia, increased fractional excretion of uric acid and increased risk of exercise-induced acute renal failure. Single nucleotide polymorphisms in the *SLC2A9* genes have also been associated with gout, coronary artery disease, and myocardial infarction [5].

All 14 GLUT members share common structural features such as 12 transmembrane helices, cytoplasmic amino and carboxy termini, and an N-linked glycosylation site, although the glycosylation site varies across the family. Regarding GLUT9, two isoforms, SLC2A9a and SLC2A9b, have been described encoding the two proteins hGLUT9a and b that differ only by the first 29 residues of the N-terminal domains [6]. GLUT9a is expressed ubiquitously, while GLUT9b is restricted to the main organs involved in urate transport, such as liver and kidney [7].

GLUT9-mediated urate transport has been characterized. It is independent of sodium, chloride and anions, but is voltage-dependent and currents have been recorded at physiological pH. Altogether, the data provided so far are compatible with a transport model in which GLUT9 is a uniport, without having formally excluded all other possibilities.

The question remains how similar GLUT9 is to other members of the GLUT family. Like GLUT1-4, it was demonstrated that GLUT9 is capable of transporting monosaccharides when heterologously expressed in *X. laevis* oocytes. However, this finding is controversial as the rate reported is very low and *in-vivo* transport is unlikely [8]. Pharmacologically, GLUT9 is not inhibited by cytochalasin B, a powerful non-competitive inhibitor of glucose transport across the GLUT family. Furthermore, the group of Thorens reported that deletion of Glut2 in the liver blocked glucose uptake in murine hepatocytes even with high expression levels of Glut9 present, indicating Glut9 is not responsible for physiological uptake of glucose [9]. It is apparent that the relationship between GLUT9 and other members of the glucose transporting family is quite complex and further work must be done to advance our understanding of this atypical transporter.

Human membrane proteins are difficult to express for subsequent purification and structural analysis. However, the knowledge of a structure yields significant advancements toward an understanding of detailed mechanism of substrate binding and transport. Modeling could reveal distinct pockets required to facilitate substrate movement as well as potential binding sites for modulators leading to pharmacological advancement and clinical development. Little was known about the detailed structure of GLUTs, in particular hGLUT9. Some biochemical approaches, such as cysteine scanning, did reveal a basic topological model of GLUT transporters [10]. Additionally, a homology model was generating based on the glycerol-3-Phosphate antiporter (PDB ID: 1PW4) [11]. Unfortunately, the template structure shares little relevance to hGLUT9 as the sequence alignment coverage represents only short regions with a total of 31% of the protein. Of the 31% aligned, the homology had only 36% identity with hGLUT9 indicating the need for further information before structure-function studies could be initiated. The 2012 publication of a high-resolution crystal structure of the bacterial homologue, XylE was an informative breakthrough allowing structural insights into the glucose transport. XylE transports the monosaccharide D-xylose and in the original work, Sun et. al established models for the glucose transporting members of the SLC2 family, hGLUT1-4. However, even with publication of the XylE structure, it is unclear if other members of the hGLUT family, and in particular hGLUT9, could be modeled in the same fashion as GLUT1-4. Based on homology, and substrate specificity, GLUT9 diverges from the rest of the group. In this work, we establish a new homology-based model for hGLUT9, based on the crystal structure of XylE. We further detail the functional expression and purification of hGLUT9 in *X. laevis* oocytes. Finally, we utilized our purified protein to generate a single particle reconstruction of isolated monomeric human GLUT9. As expected, the low-resolution reconstruction merges well with the homology-based model, providing initial support for the use of our predictive model as a tool for subsequent structure-function studies. We conclude by demonstrating the potential of the homology-based model to uncover the binding pocket for hGLUT9's unique substrate urate.

## Materials and Methods

### Ethics Statement

All animal experiments were in accordance with the Swiss animal welfare law and were approved by the local Veterinary Authority Bern (Veterinäramt Bern; Permit Number: BE26/12).

### Materials

We purchased Superose 6 10/300 GL from Amersham Biosciences (#17-5172-01, GE Healthcare Europe, Glattbrugg, Switzerland); sodium dodecyl sulphate (SDS) from Sigma-Aldrich (#L6026, St. Louis, MO, USA); n-Dodecyl-ß-D-Maltopyranoside (DDM) from Affymetrix (#D310, St. Clara, CA, USA). Human Rhinovirus (HRV) 3C protease was purchased from AG Scientific (#H-1192, San Diego, CA, USA).

### Computational Analysis

Amino acid identity and relationships between bacterial and human SLC2 homologue were performed using the ClustalW2.1 program [12] with the BLOSUM matrix. The NCBI reference sequence numbers used was NP_418455.1 (XylE), NP_006507.2 (SLC2A1), NP_000331.1 (SLC2A2), NP_008862.1 (SLC2A3), NP_001033.1 (SLC2A4), NP_064425.2 (SLC2A9a) and NP_00100190.1 (SLC2A9b). Phylogenetic tree generation of SLC2 family members was based on the results from the BLOSUM matrix within SeaView 4 software (distance measurement method using BioNJ algorithm with Poisson parameter) [13]. EasyModeller 2.1 [14] was used to build the predicted structure of hGLUT9 from its FASTA sequence NP_064425.2 using the crystal structure of the bacterial homologue XylE (PDB ID: 4GBY) as a template. The Orientation of Proteins in Membrane Database (PPM Server) calculates rotational and translational positions of hGLUT9 protein within membrane bilayer using their predictive 3D-structure (PDB coordinate file) as an input [15].

### Human GLUT9b (SLC2A9) Sub-Cloning

cDNA from hGLUT9b (*Gene ID: 56606*) was purchased from Origene and sub-cloned by PCR into the pMJB08 expression vector using forward primers 5′ CAT TTC TCG AGA TGA AGC TCA GTA AAA AGG ACC GAG G′ and reverse primers 5′ TTA ATT CTA GAT TAA GGC CTT CCA TTT ATC TTA CCA TC 3′. PCR products were digested with the restriction enzymes *XhoI* and *XbaI*, and ligated into the vector. The recombinant protein was tagged with an N-terminal, 10xHis, FLAG tag, and human rhinovirus 3C protease (HRV 3C) cleavage site known as PreScission followed by a hemaglutinin (HA) tag [16].

### *Xenopus laevis* Expression

Oocytes were prepared and injected at stage V-VI with 20 ng of cRNA encoding SLC2A9b. In brief, animals were anesthetized by cooling at 4°C with tricaine mesylate (3-aminobenzoic acid ethyl ester, methane sulfonate salt, 150 mg/l). Small pieces of ovary were isolated in sterile Barth solution (10 mM HEPES pH 7.4, 88 mM NaCl, 1 mM KCl, 2.4 mM NaHCO_3_, 0.82 mM MgSO_4_, 0.33 mM Ca(NO_3_)_2_, and 0.41 mM CaCl_2_, supplemented with 50 µg/ml gentamycin). Oocyte defolliculation occurred in calcium-free modified Barth's solution with 3 mg/ml collagenase NB4 (SERVA Electrophoresis, Heidelberg, Germany). Isolated oocytes were then incubated overnight in standard Barth's solution. After 24 hours, injection of cRNA was performed in at least 1,500 oocytes using an automated injection device (Roboinject, Multi Channel Systems, Reutlingen, Germany). Oocytes were maintained in Barth's Solution for 3 days at 18°C to maximize expression for functional and biochemical studies.

Oocytes were solubilized and crudely homogenized using a 100 µl pipette tip with ice-cold RIPA lysis buffer as described previously [17]. After 45 min on ice, samples were centrifuged at 15,000 *g* (15 min at 4°C). Only the supernatant below the yolk was taken for SDS-PAGE and Western blot analysis.

### Protein Deglycosylation

For deglycosylation experiments, oocytes were solubilized as described above with modified SDS-free RIPA buffer (20 mM Tris·Cl pH 7.4, 150 mM NaCl, 0.5% DDM, 1% NP-40, 0.5% DOC) supplemented with 1 mM PMSF and EDTA-free protease-inhibitor cocktail. Following solubilisation, the manufacturer's protocol was followed for enzymatic deglycosylation (The Protein Deglycolsylation Mix, NEB, #P6039, Ipswich, MA, USA). In brief, 40 µg of total protein were incubated in glycoprotein denaturing buffer for 30 min at 37°C. Solution was diluted in G7 reaction buffer with NP-40 and PNGase was added for 1 hr at 37°C. Samples were analyzed by SDS-PAGE and Western blot.

### Plasma-Membrane Biotinylation

A minimum of 20 hGLUT9-injected oocytes and 20 water-injected oocytes were washed twice in phosphate buffered saline (PBS). Oocytes were subsequently incubated in PBS supplemented with 1.5 mg/ml LC-Sulfo-NHS Biotin (Molecular BioScience, #00598, Boulder, CO, USA) for 60 min at 4°C under gentle agitation. Oocytes were washed by 3 successive baths of PBS containing 100 mM glycine to stop the reaction, followed by a final rinse in PBS to remove excess glycine. Oocytes were transferred to pre-cooled Eppendorf tubes with ice-cold RIPA lysis buffer added to a final volume of 1 ml. Cells were crudely homogenized and incubated for 120 min at 4°C under rotation. In parallel, a final volume of 37 µl of streptavidin beads (Thermo Scientific, #20349, MA, USA) were prepared washed and equilibrated into 1 ml of RIPA lysis buffer for each sample to be tested. Beads were centrifuged at 9,000 *g* and the RIPA supernatant was removed. After the 120 min sample incubation, lysates were centrifuged at 15,000 *g* for 15 min at 4°C. Equal amounts of supernatant (approximately 500 µl) were transferred to the previously prepared streptavidin beads. Samples were incubated O/N at 4°C under rotation. Samples were then centrifuged at 9,000 *g* for 1 minute at 4°C. After incubation, 50 µl of the supernatant was retained corresponding to the cytoplasmic fraction. Beads were subsequently washed 3 x times in 1 ml of RIPA buffer. Beads were spun down and excess supernatant was removed. Protein was eluted from the beads by addition of 100 µl of 2X SDS-loading buffer containing DTT and membrane fraction was heated for 15 min at 95°C. Centrifuge beads for 1 min at 4°C at 9,000 *g* and load 25 µl of supernatant for SDS PAGE and Western blot analysis.

### SDS-PAGE and Western Blotting

Protein concentrations were determined using a BCA protein assay kit (Thermo Scientific, MA, USA). For SDS-PAGE, samples were prepared on 8% polyacrylamide gels and transferred to PVDF membranes (Amersham). Monoclonal HA-epitope antibodies (α-HA) were purchased from Sigma-Aldrich (#H3663, St. Louis, MO, USA) and used at a final dilution of 1∶1000. Immunodetection was performed using goat anti-mouse IgG (1∶3000) (H+L) horseradish peroxidase (Bio-Rad, #172-1011, Hercules, CA, USA) with an enhanced chemiluminescence detection system (Amersham Biosciences, GE Healthcare Europe, ECL+ #RPN2106, Glattbrugg, Switzerland).

### Electrophysiology

Oocytes were impaled with two electrodes filled with 3 M KCl, and their membrane potentials were maintained at −60 mV throughout the experiment. All recordings were performed at 18°C and superfused with OR2 medium (5 mM HEPES pH 7.4, 82.5 mM NaCl, 2.5 mM KCl, 1.8 mM CaCl_2_ and 1 mM MgCl_2_,). Currents were recorded using an automated process equipped with standard two-electrode voltage clamp (TEV) configuration (Multi Channel Systems, Reutlingen, Germany). Data was analysed using Excel (Microsoft, Redmond, WA) software. Uric acid was prepared directly before usage in the recording medium to obtain the desired test concentrations. All experiments were carried out using three or more *X*. *laevis* oocytes.

### Total Membrane Preparation

Oocytes were homogenized with a Teflon-glass homogenizer in lysis buffer containing 50 mM Tris·Cl pH 8.0 supplemented with protease inhibitor cocktail (Sigma-Aldrich, #S8830, St. Louis, MO, USA) and 1 mM PMSF and fixed on rotator at 400 rpm (Homogenisator Potter S, B. Braun, Melsungen, Germany). Homogenates were centrifuged at 1,500 *g* for 15 min at 4°C to discard nuclear and cellular debris. Supernatant was centrifuged at 150,000 *g* for 1 hr at 4°C to pellet down total membranes. Membranes pellets were resuspended in lysis buffer with 1 M NaCl to solubilize contaminant membrane associated proteins (e.g. vitellogenins) and further centrifuged at 150,000 *g* for 1 hr at 4°C [16]. The final membrane pellet was resuspended in 20 mM Tris·Cl pH 8.0, 300 mM NaCl and 10% (v/v) glycerol at about 20 mg/ml and stored at −80°C.

### Ion Metal Affinity Chromatography (IMAC) Purification of hGLUT9b and Size Exclusion Chromatography (SEC)

Membranes suspensions were diluted to 2.5 mg/ml final concentration in 50 mM Tris·Cl pH 8.0, 1 M NaCl supplemented with protease inhibitor cocktail and PMSF. DDM (D/P ratio  = 15) was used to solubilized membranes at 4°C for 2.5 hrs under gentle rotation. The lysates were centrifuged at 5,000 *g* for 5 min at 4°C and supernatant was loaded on a cobalt column and incubated overnight (14 mg total protein/ml settled gel, equilibrated with 20 mM Tris·Cl pH 8.0, 300 mM NaCl, 0.1% DDM with 5 mM Imidazole). The resin was washed 2 times with equilibration buffer containing 40 mM imidazole (10 resin volumes). SLC2A9b was eluted by incubation with Human Rhinovirus (HRV) 3C protease at 70 µg/ml for 2 hrs at 4°C under constant agitation. After elution, the protein was concentrated to final volume of 70 µl using an Amicon 50 kDa cut-off centrifugal filter (Millipore, #051382, MA, USA). A Superose 6 10/300 GL gel filtration column was connected to ÄKTA*prime* workstation (GE Healthcare) and equilibrated with 20 mM Tris·Cl pH 8.0, 150 mM NaCl, 0.1% DDM. The solution was filtered at 0.22 µm and sonicated in a water bath for 5 min to minimize bubble formation. Sample was eluted with equilibrium buffer at a flow rate of 0.3 ml/min. Absorbance at 280 nm was monitored with in-line UV-detector and all fractions of 500 µl were collected and analysed.

### Negative-Stain TEM and Single Particle Reconstruction (SPR)

Negative-stain TEM and grid preparation was performed as described [17]. In brief, solubilized hGLUT9b (15 µg/ml) was adsorbed for 10 seconds to parlodion carbon-coated hydrophobic copper grids. Grids were washed in ddH_2_0 and negatively stained with 0.75% (w/v) uranyl acetate. Electron micrographs were recorded at a magnification of 110,000× on a Morada CCD camera from OLYMPUS where pixel size was 3.092 Å. The Philips CM-12 electron microscope operated at 80-kV acceleration voltage.

Single Particle Reconstruction (SPR) was performed using EMAN2 (Electron Micrograph ANalysis) open-source suite programs [18]. Digital TIFF images (2970×2100) were recorded in 16-bit using the software iTEM (OLYMPUS) under constant focus and astigmatism corrections. Standard EM parameters were used to capture micrographs. The images were obtained free of drift, vibration and astigmatism, with slight under-focus controlled by the iTEM software during live acquisition. A detailed process of the subsequent SPR is described below.

i. Particle selection (e2boxer.py)

Contrast level were adjusted by Photoshop CS suite for each micrographs and saved in 8-bit. Electron micrographs were import in EMAN2 suite. In EMAN2, all program are executed using the built-in workflow GUI (e2workflow.py). We apply a number of common filters to the data before importing such as Edge nom thought e2workflow.py program. All micrographs are saved as “MRC” (Medical Research Council) files. Particles were selected with box dimension 84×84 pixels as 7056-dimensional vector using semi-automatic picking function by Swarm mode (algorithm uses a trainable heuristic based approach) in e2boxer.py program. The results were manually verified, and false positives were eliminated, at this step of the process image quality weight can be addressed 0 to 4. When all particles are selected output can be written with box coordinates. Images were normalized by the normalize.edegemean option. Output images were saved in default format “BDB”files used for processing in the workflow interface.

ii. CTF and phase flipping corrections (e2ctf.py)

The aim of SPR is to generate the “true” 3D structure of a macromolecule based on its 2D projections. Inherent of contrast transfer function (CTF) and the envelope function of the electron microscope, the projections observed are not reflective of the real projections of the electron density of the specimen. CTF is a mathematical representation of the imaging process in the TEM, examined in reciprocal space. Begin with selecting the particles intended to generate the CTF parameters using the following steps: i/Autofit, ii/manually fine-tune parameters for a few sets at different defocuses iii/generate a structure factor using these sets iv/re-run autofit v/manually check the fitting results. In practice, 2D power spectra and 1D averaged power spectrum of the boxed out particles from each single image is used to characterize the CTF with three parameters: defocus, B-factor and %AC (Amplitude Contrast). The particle set is built from phase-flipped output. The Phase-flipping corrections simply consist of multiplying the Fourier transform of each particle image by -1 over the appropriate frequency ranges.

iii. Reference-free class averages (e2refine2d.py)

In this step, the strategy is to sort raw particles presenting the same orientations into different groups based solely on their 2D projection characteristics. A set of representative class-averages is generated and only used to assess the structural variability of the specimen and to create an initial 3D-model. The result is a reduction of the noise level to give a shape more detailed from specific or “class” angles of the specimen corresponding to the different views.

iv. Initial model building (e2initialmodel.py)

This program called e2initialmodel uses class-averages produced to create few initial models classified by quality for use in further refinement. Based on the crystal structure of XylE, hGLUT9 was assumed to be asymmetrical. Therefore, we utilized the Asymmetrical C1 parameter within the program to generate the initial model. The 3D-model generated from the class averages was used to accommodate the predicted homology-based model of hGLUT9 and for further refinement within SPR.

v. Refinement and Resolution (e2refine.py, e2eotest.py, e2resolution.py)

Based on the initial model, back projections in all obtained orientations are generated and computationally compared to the original 2D-projections to identify similarities among the individual classes. Sets of more similar projections are iteratively aligned and averaged. Class-averages, for which the orientation in known, as defined by the initial model, are used to build a new, more refined, 3D-model. Three iterations and the generated Fourier Shell Correlation curve at 0.5 were used to define the resolution of the reconstruction at 23 Å.

## Results

### Predicted Structure of Human SLC2A9 (hGLUT9)

**Figure 1:**
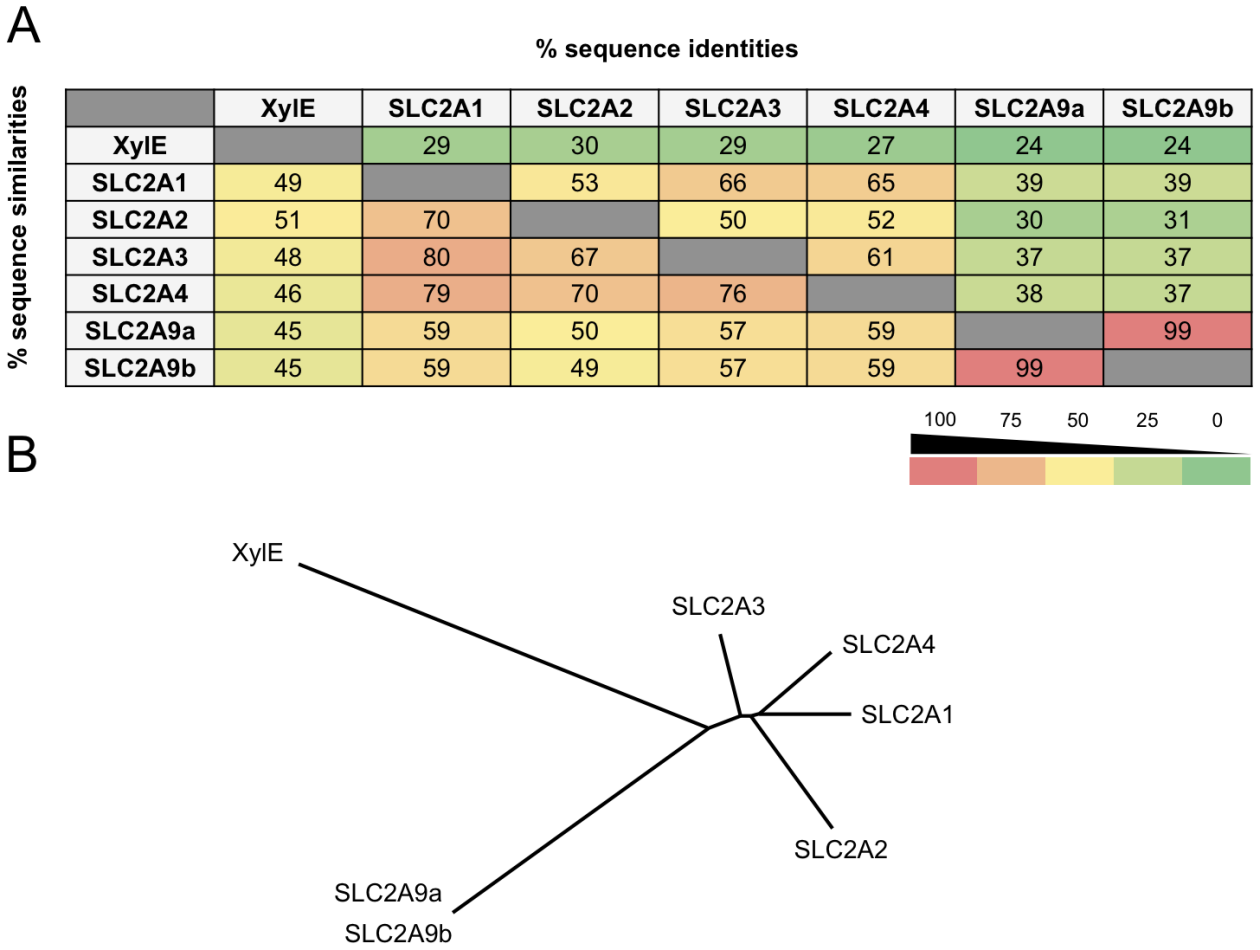
Phylogenetic tree and sequence relationships of the SLC2 family members. (A) Bacterial XylE sequence identity and similarity heat map comparing various members of the human GLUT transporter family. SLC2A9a and b share the least sequence similarities and identities when compared to SLC2A1–4 and are further separated from the bacterial homologue XylE. (B) Phylogenic tree representation of the relationship between GLUT family members.

Recently, JIANG et *al.* published the high-resolution structure of the bacterial SLC2 homologue XylE co-crystallized with its substrate D-xylose. From these results, (JIANG) reconstructed the human SLC2A1 (GLUT1) protein structure from sequence homology validated by further mutagenesis experiments [19]. Due to the diversity present within the SLC2 family of human proteins, it was not known whether additional members could be reconstructed in the same manner. To determine the relationship and sequence homology between different SLC2 members and their bacterial homologue XylE, a “heat map” matrix and associated phylogenic tree was generated (**Figure 1A**) based on the NCBI BLAST alignment shown in **Figure S1**. SLC2A(1–4) have high sequence identity and similarity averaging 58 and 74%, respectively. With respect to XylE, SLC2A(1–4) share 29% identity and 49% sequence similarity. However, SLC2A9 isoforms share a more distant relationship to both SLC2A(1–4) and XylE with average sequence identities and similarities at 36/56% and 24/45%, respectively. The relative distances between homologues is better represented by the phylogenic tree analysis (**Figure 1B**) showing clear separation of SLC2A9 from other human GLUT proteins.

**Figure 2:**
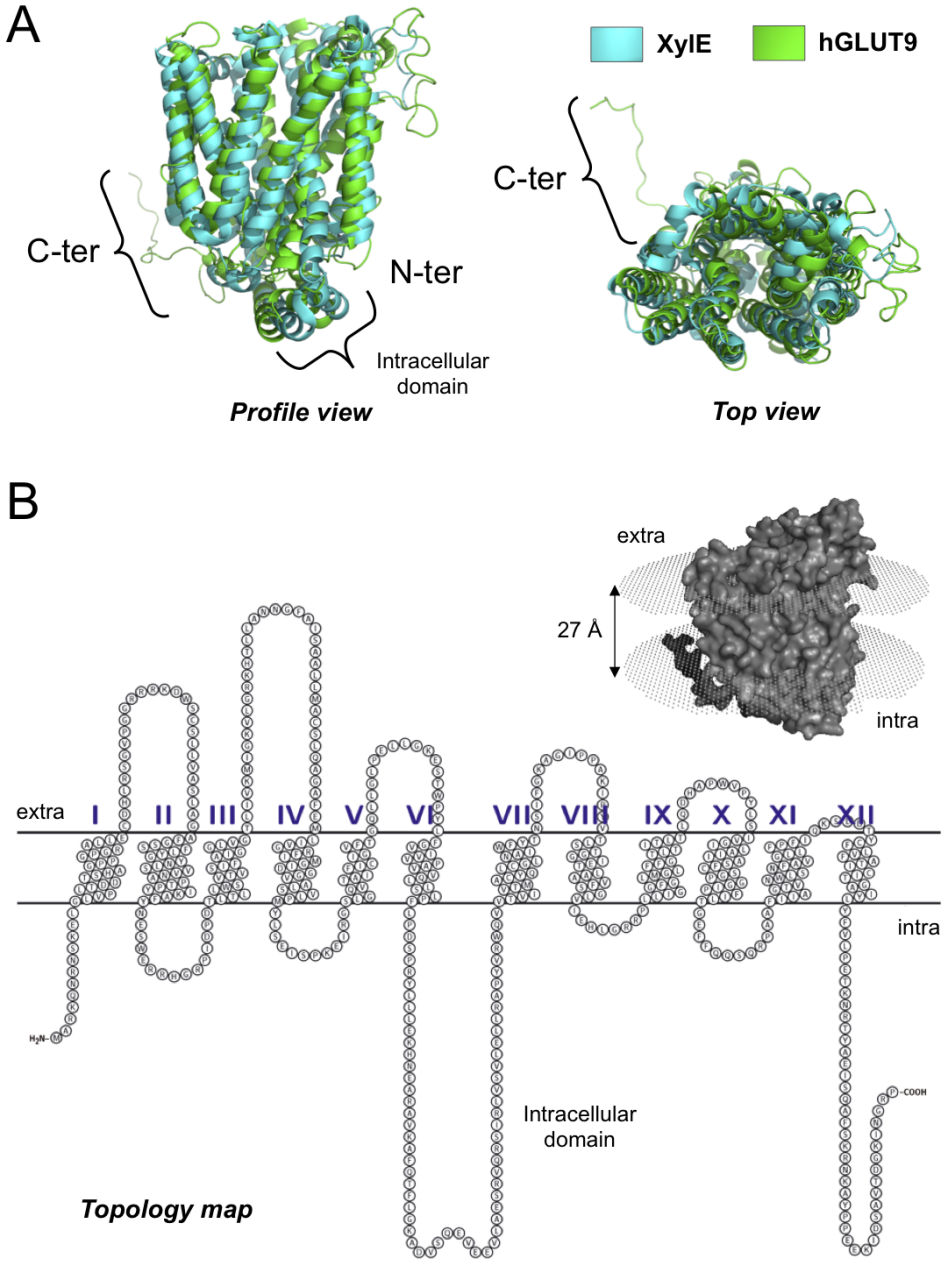
Homology-based modeling of hGLUT9 structure. The 3D-structural model of hGLUT9 is generated from sequence alignment with the bacterial homologue XylE. (A) Merge of hGLUT9 (green) and XylE (blue) 3-D structures: visualized with PyMOL v0.99 software. Even with the decreased homology of hGLUT9 versus GLUT1–4, the putative topological model corresponds to the one of XylE. (B) Putative 3D-structure generated by EasyModeller 2.1 embedded within a simulated bilayer as calculated from hydrophobicity charge analysis. Representation of the putative model is translated into a 2-D topological map. Note that only trans-membrane helices are represented, with exclusion of the large cytoplasmic helices between transmembrane VI and VII.

In order to determine if it was possible to derive an accurate structure of hGLUT9, a predictive homology-based model structure was designed enabling subsequent validation using low-resolution structural, biochemical, and functional analysis. The program EasyModeller 2.1 was used to construct the predicted structure of hGLUT9 from its FASTA sequence NP_064425.2 using the crystal structure of a bacterial homologue named XylE (PDB ID: 4GBY). The structure obtained is similar to the bacterial homologue composed of 12 transmembrane segments (TMs) and of one intracellular domain (**Figure 2A**). The human protein contains an additional unfolded long C-terminal domain (C-ter) not present in the bacterial homologue. From the generated hGLUT9 structure, a schematic topology representation depicting transmembrane helices was realized using OPM Database (**Figure 2B** **, insert**). Depth/hydrophobic thickness is 26.8±2.2 Å with ΔG_transfer_ = −55.5 kCal/mol and tilt angle  = 6±1°. The TexTopo program was used to represent topology of the protein showing only transmembrane helix part: TM1 (G13-E35), TM2 (A64-Y89), TM3 (T105-G124), TM4 (L167-M187), TM5 (S200-T215), TM6 (F235-F251), TM7 (V318-T339), TM8 (T357-V374), TM9 (L383-T403), TM10 (I416-T438), TM11 (F451-I472) and TM12 (Y478-L495) in **Figure 2B**.

### Expression Analysis of GLUT9 in *Xenopus laevis* Oocyte System

The *X. laevis* oocytes expression system was chosen to express hGLUT9 due to the unique capabilities that allow for high protein expression and moderate-throughput functional characterization within the same expression vector. The goal was to begin validation by functional expression, purification and reconstruction of hGLUT9 and comparison to the homology-based model predicted from XylE.

**Figure 3:**
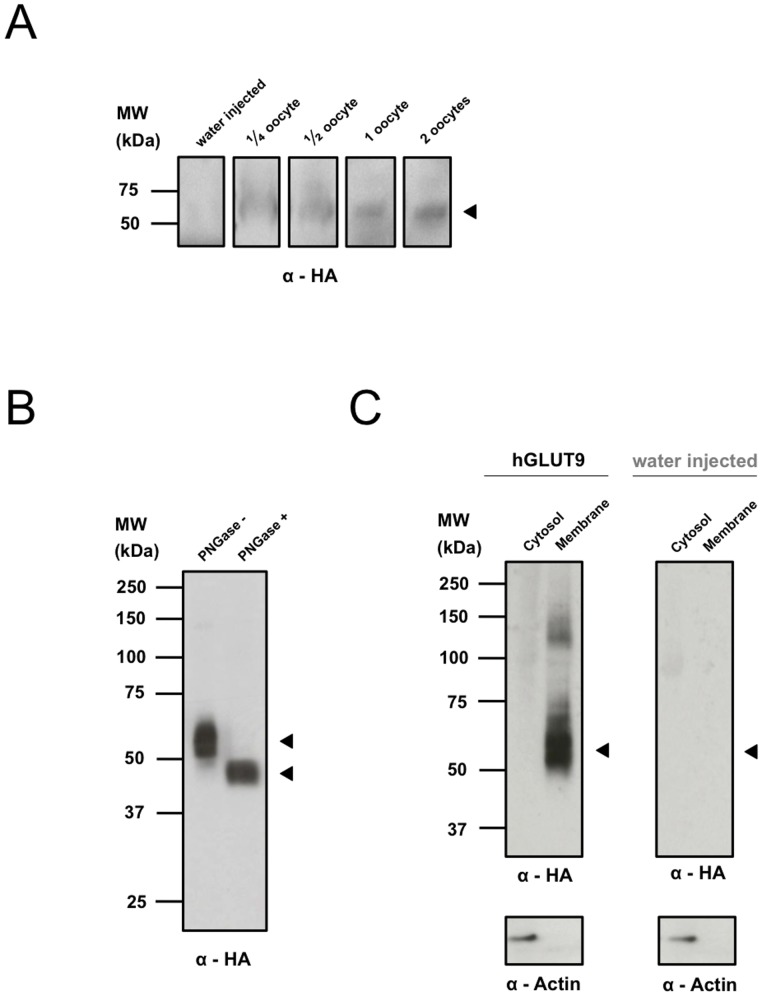
Surface expression of hGLUT9b in *X. laevis* oocytes. (A) Expression level of hGLUT9b by Western blot analysis. (B) Surface expression of hGLUT9b as determined by deglycosylation analysis using PNGase of fully denatured DDM based total lysate. (C) Surface biotinylation and pull-down reveals a highly enriched hGLUT9b surface membrane fraction.

We sub-cloned the cDNA of SLC2A9b into a vector containing an N-terminal purification tag. cDNA was *in vitro*-transcribed into cRNA and injected into *Xenopus laevis* oocytes. Western blot analysis of membrane preparations correlating to the amount of protein isolated from individual or fractional oocytes demonstrated that GLUT9 is highly expressed in oocytes with protein levels detected in as low as ¼ of an oocyte (**Figure 3A**).

To validate the surface expression of GLUT9 two independent protocols were performed: surface biotinylation to label and pull down only protein expressed on the plasma membrane and protein deglycosylation analysis, to determine if there is a Western blot shift due to enzymatic removal of extracellular glycans. **Figure 3B** demonstrates a 10 kDa shift for hGLUT9 treated with the non-specific deglycosylating enzyme PNGase indicating proper protein folding within the ER and subsequent surface expression. This observation was confirmed through membrane biotinylation pull-down in **Figure 3C**. Water-injected and hGLUT9-injected oocytes were surface-labelled with biotin. Cells were lysed and total protein was incubated on streptavidin beads. Protein isolated from the beads contains only the surface membrane fraction and expression is clearly enriched in hGLUT9-injected oocytes, whereas the cytosolic supernatant fraction shows clear actin binding but little to no hGLUT9, indicative of high-levels of surface protein expression.

### Functional Analysis of hGLUT9 in *Xenopus laevis* Oocyte System

**Figure 4:**
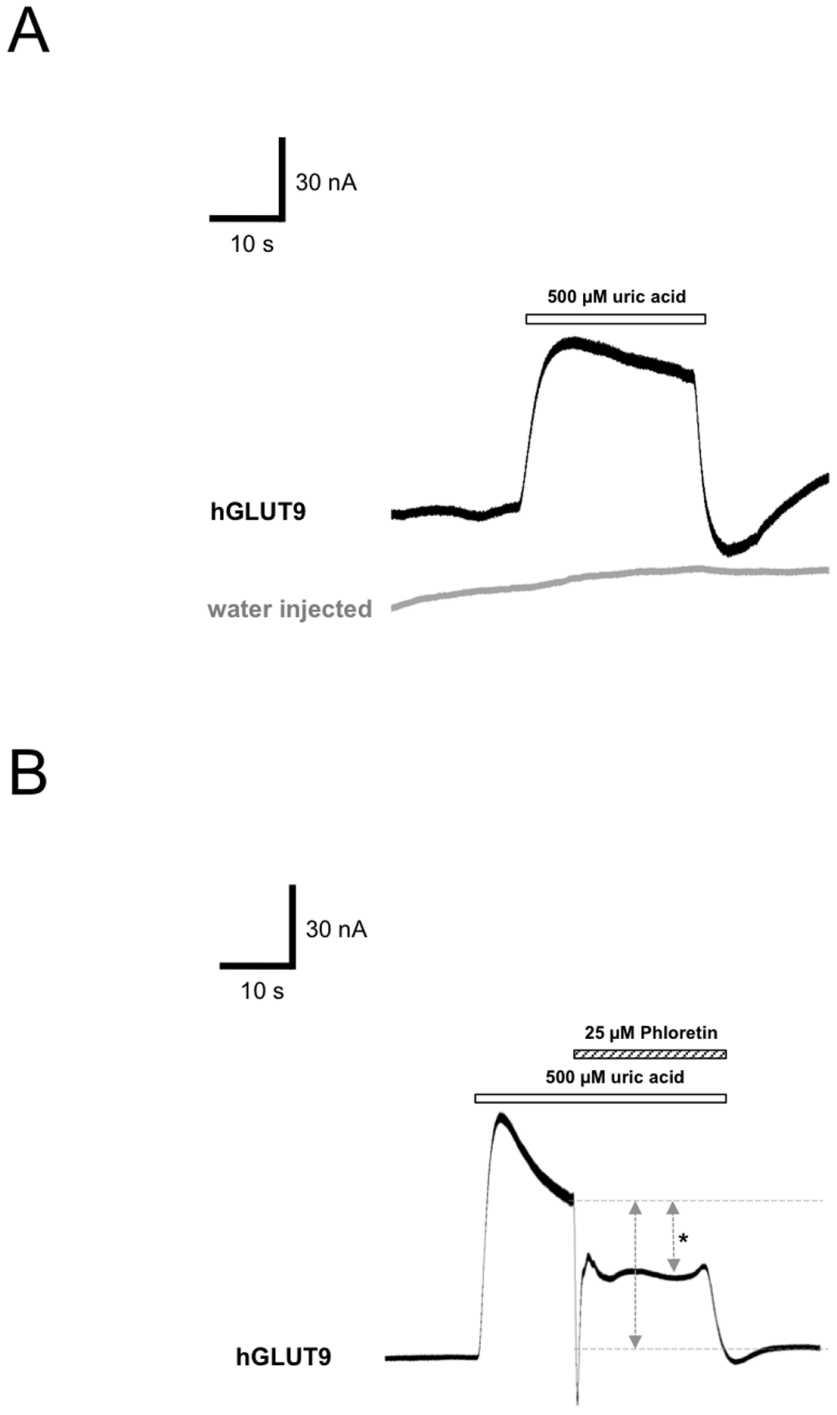
Functional analysis of hGLUT9b in *X. laevis* oocytes. (A) Water injected controls and hGLUT9b expressing oocytes were clamped at −30 mV and exposed for 30 sec to 500 µM urate containing OR2 medium. Current evoked by urate averaged 70+/−10 nA (n>20). (B) Same experiment as outlined above, but with application of the non-specific GLUT9 inhibitor, phloretin. Oocyte were clamped at −30 mV and exposed for 15 sec to 500 µM urate followed by 20 sec of 500 µM urate +25 µM Phloretin. Phloretin inhibited uric acid current by 45±4% (n = 3).

Functional surface expression was determined using an automated two electrodes voltage clamp system (HiClamp). Oocytes were exposed to 500 µM uric acid for 20 sec, water injected oocyte as negative control, was measured (n = 5). The water injected oocyte showed no effect to uric acid, whereas the cRNA injected oocytes showed an outward current of 80±8 nA while the oocyte was clamped at −60 mV (**Figure 4A**). The current behaviour was also measured for untagged protein indicating that the N-terminal modification had no impaired on the expression level or on functional level (data no shown). We also observed inhibition of SLC2A9 activity through application of 25 µM phloretin, a non-specific GLUT9 inhibitor (**Figure 4B**). GLUT9 was activated with 500 µM uric acid followed directly by application of phloretin in the presence of uric acid. This resulted in a reduction of 45%±5% of transporter activity (n = 8) corresponding nicely to previously described radioactive uptake studies [3]. Water-injected oocytes displayed no alterations of current activity in the presence of phloretin (Data not shown).

### Purification of GLUT9 by IMAC and SEC

**Figure 5:**
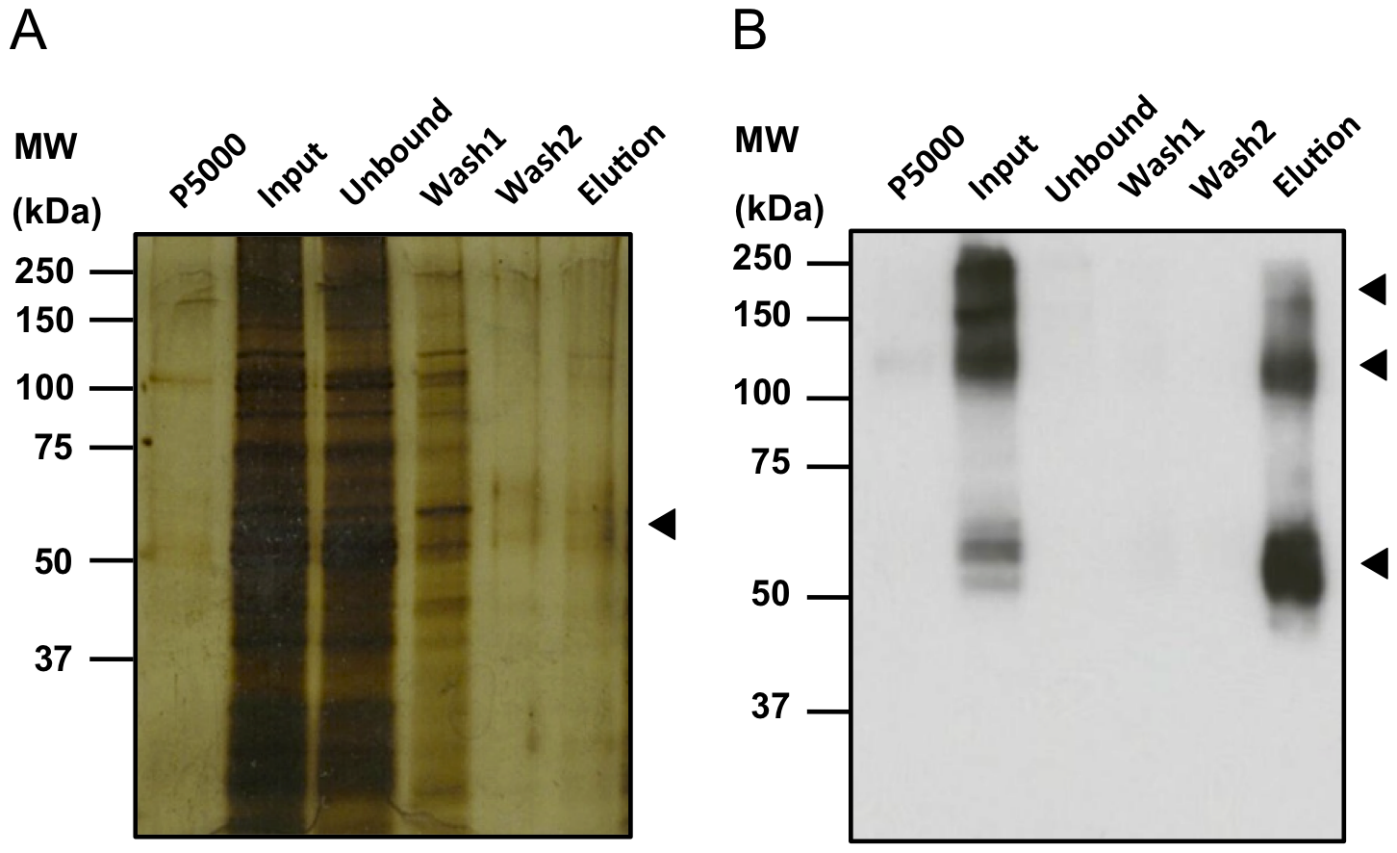
SDS-PAGE, silver staining and Western blot analyses hGLUT9b purification after IMAC from *X. laevis* oocytes membrane preparation. (A) Silver-stained and (B) Western blot SDS/polyacrylamide gels show that recombinant human GLUT9b runs at ∼60 kDa, corresponding to the expected molecular weight. Oligomers are observed at ∼120 and 200 kDa. First line corresponds to the pellet fraction at 5,000 *g* after membrane solubilization (P5000) and the supernatant was loaded on the IMAC column (Input). The Western blot using anti-HA shows that all hGLUT9b was extracted and solubilized from the membrane fraction. The three following lanes correspond to the unbound and washed fractions and demonstrate that hGLUT9b binds to the column. The protein was eluted using HRV 3C protease at the PreScission site.

After demonstrating that hGLUT9 is functionally active at the surface membrane of oocytes, work was performed to optimizing the purification methods required for further structural studies with a focus on maintaining physiological conformation. The correct choice of detergent is crucial to maintain a functional representative and stable form of solubilized membrane proteins. DDM, a mild non-ionic detergent, commonly used for the solubilisation of GPCRs [20] was shown to completely extract and solubilize GLUT9 from membrane preparations in silver staining (**Figure 5A**). P5000 represents the insoluble membrane fraction remaining after detergent extraction. This indicates that the detergent efficiently solubilized membrane proteins as the majority of the signal remains in the soluble Input fraction. The Input lane corresponds to the total soluble membrane lysate. The Unbound is the membrane lysate remaining after incubation with cobalt resin and represents total membrane lysate minus protein bound. The final Wash columns reveal the removal of non-specific low-affinity resin binding proteins and the Elution column represents the purification and extraction of hGLUT9 using the sequence specific HRV-3C protease from the cobalt resin. The use of the oocyte system for the purification of membrane proteins produces significant purified protein however the amount is typically much lower than canonical purification methods making the final elution on silver stain difficult to ascertain. Additional immune-specific techniques such as Western blotting must be used in parallel to determine the quality of the final purified protein [16], [17]. Using the same gel, Western blotting demonstrates strong signal in the elution indicating significant enrichment **(** **Figure 5B** **)**. GLUT9 was isolated using IMAC (Immobilized Metal Affinity Chromatography) as a monomer (∼60 kDa), oligomer (∼120 kDa) and aggregate (>150 kDa) from membrane preparations of 1500 oocytes.

**Figure 6:**
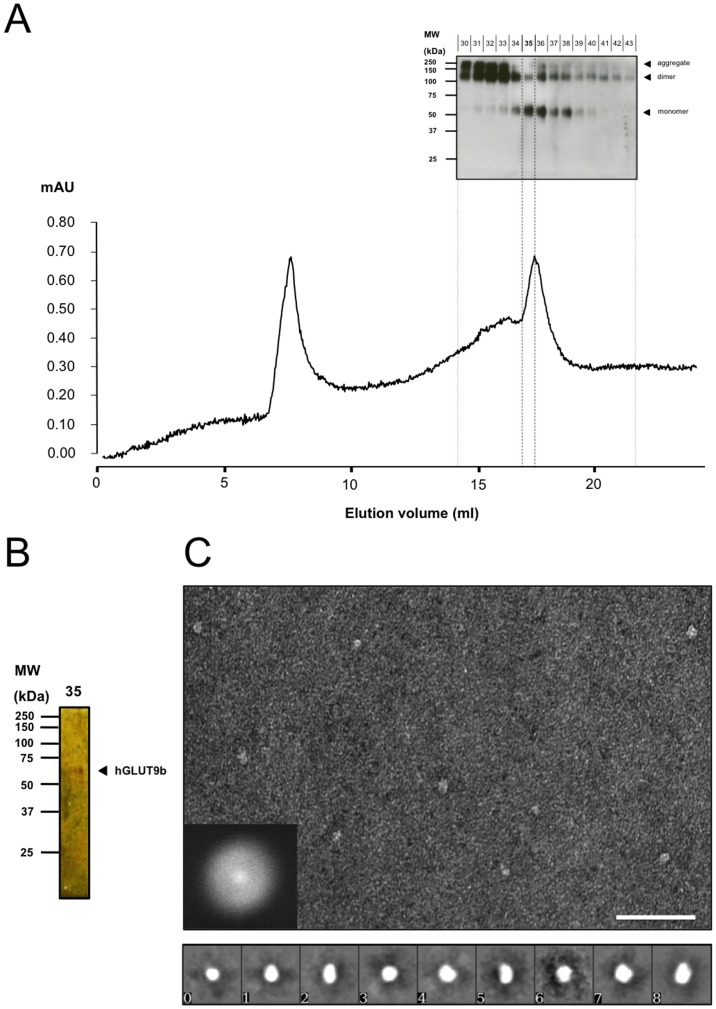
Isolation of hGLUT9b by size-exclusion chromatography with Western blot, silver stain, and single particle analysis. (A) Superose 6 gel filtration chromatography shows two peaks. The first peak corresponds to the dead volume of the gel filtration where large molecular weight proteins or aggregates do not interact with the beads and quickly pass through the column. The second peak corresponds principally to the monomeric (sharp peak, right side) and oligomeric (shoulder, left side) states. Western blot representation of fractions 30 to 43 after gel filtration showing clearly the separation of the second peak into hGLUT9b oligomers and monomer. (B) Silver-staining of hGLUT9b monomers obtained from fraction 35. (C) Micrograph of hGLUT9 particles obtained from negatively-stained TEM. Scale bar is 50 nm. Gallery representation of the 9 class-averages obtained from 1439 particles. Each representation is scaled to a 26 nm square.

Size exclusion chromatography (SEC) was performed using the eluted fractions after IMAC. The aim was to create a homogenic purified protein extract by separating the aggregated and oligomeric forms from the monomeric state (**Figure 6A**). Fraction 35 was determined to proportionally yield the most monomeric protein with minimal overlap from oligomeric states **(Figure S2A)**. **Figure 6B** demonstrates the isolated 60 kDa monomer as represented on silver stain and indicates isolation and purification of the monomeric form of hGLUT9. While the 60 kDa signal on the silver stain was relatively low, this is representative of a final protein concentration of 15 µg/ml and benefits from little to no background signal arising from oligomeric forms as well as any other additional protein contaminant.

### 3D-Reconstruction of SLC2A9b Monomers

One of the main goals of this study was to determine if the correlative model of the divergent hGLUT9 (**Figure 2**) was accurate enough for further biochemical and predictive approaches. A single particle reconstruction (SPR) approach using the monomeric state of purified hGLUT9 was used to compare our initial predicted results with low-resolution images of isolation protein of Fraction 35 from the SEC. By focusing on Fraction 35 protein negatively stained was enriched for monomeric representations of SLC2A9 (**Figure S2**).

**Figure 7:**
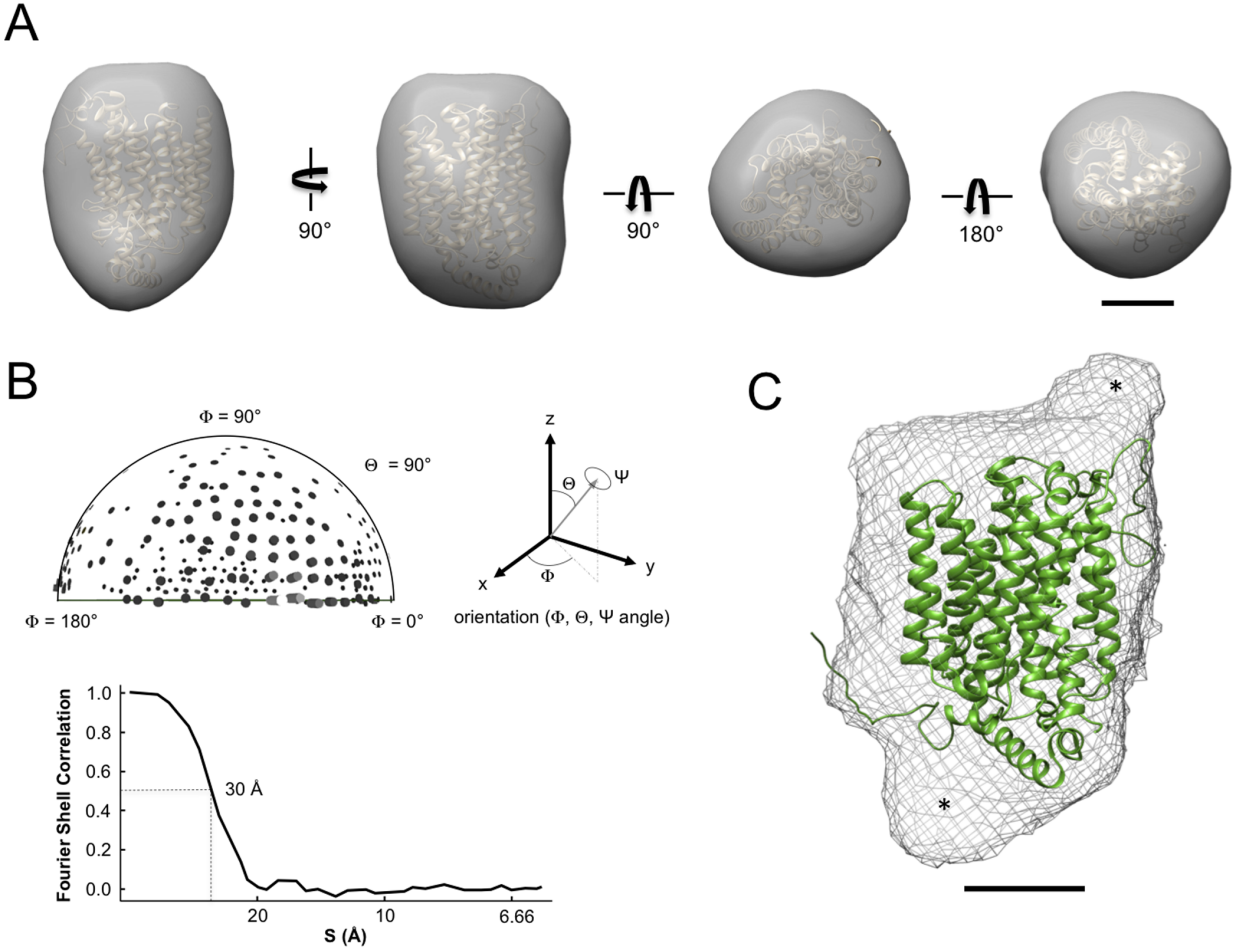
Single particle reconstruction of purified hGLUT9b monomers. (A) Different views of the initial 3D-reconstruction (gray surface) which accommodate the predicted homology-based model (gold). Scale bar is 26 Å. (B) Plot of the Euler angle distribution showing that the particles adsorb to the carbon film in random and uniformly distributed orientations. The initial model was determined to have a resolution of 30 Å as estimated using a Fourier Shell Correlation curve. The refined model improved the resolution to a final 23 Å (C). The final refined model showing the original homology based-structure within the density map of the observed hGLUT9 particles. Alignment showed significant overlap with two areas noted by an asterisk corresponding to unstructured domains.

The monomeric SLC2A9 3D-model was generated by single particle reconstruction (SPR) utilizing EMAN2 suite. The aim of “single particle” image processing is to obtain a 3D-reconstruction of a macromolecule from a large set of 1,439 particle images obtained from TEM. The single particle reconstruction approach is based on the assumption that the sample is homogeneous and that particles are randomly oriented on EM grid, generating different projections. Micrographs of a field of negatively stained SLC2A9 monomers were used to generate particles for SPR (**Figure 6C**). The quality of the micrograph is demonstrated by the inset FFT. A particle set was isolated from the micrographs with a total of 9 class averages (0–8), representing different orientations (front, top and side) of the particles (**Figure 6C**). From the class averages, an initial model was generated (**Figure 7A**). The larger projections were approximately 85×65 Å and maintained ovular shapes. The v-shaped projection and more rectangular projection and were aligned to represent the side views of hGLUT9, respectively (**Figure 7A** **(** ***i*** **) and (** ***ii*** **)**). The smaller, circular projections represent the top and bottom view, respectively (**Figure 7A** **(** ***iii*** **) and (** ***iv*** **)**). Presence of the particles in different orientations, as represented by the Euler plot (**Figure 7B**), indicated that there was even distribution of hGLUT9b monomers on the support film with only minor insufficiency between Φ90**°** and Φ180° due to the limitations inherent with negative staining and the glow-discharge techniques. After three iterative refinement process, a 3D-model at low-resolution was generated to assess to the validity of our predictive model. Based on the Fourier Shell Correlation, FSC = 0.5, we tested the resolution of our models using the script *e2resolution* within EMAN2 and obtained a 30 Å result for the initial model and 23 Å resolution after further refinement **(** **Figure 7B** **)**. An overlay of the refined 3-D reconstruction model generated from the TEM micrographs matches reasonably with the predicted homology-based structure of hGLUT9 as shown in **Figure 7C**. To aid in the interpretation of the model, we used the “Fit in Map” function within Chimera to align the predicted structure with the generated density map. Notably, two regions, (labeled “*”) obtained after single particle reconstructions, were not fill by the predictive structure. This is due to the rigid fitting method employed in the predictive crystallographic structure not representing the potential dynamics of the protein structure that involve movement of secondary domains.

## Discussion

In the present study, we developed a model of the human uric acid transporter GLUT9 and established protocols for the expression, purification, and functional analysis of hGLUT9 using the *X*. *laevis* oocyte expression system. The aim of this work was two-fold. The first goal was to detail procedures for functional expression of human GLUT9 protein to allow subsequent biochemistry and structure-function analysis. This work is of considerable interest as it is currently unknown why GLUT9 is unique amongst GLUT family members in its ability to transport uric acid. Physiologically, humans have one of the highest serum uric acid levels among mammals, and a greater understanding of the structure function of one of the main uric acid transporters may lead to advancements in the treatment of gout as well as the determining the role uric acid may play in neuro-protection and oxidative stress. The second goal was to determine if GLUT9, which shares the least homology with other members of the SLC2 family, could be sufficiently modelled from the recently published bacterial homologue XylE. The modelling of hGLUT9 is a prerequisite for the determination of a putative binding pocket for uric acid, but due to a lack of sequence identity and the phylogenic distance of hGLUT9, we developed a low-resolution single-particle reconstruction of the human protein purified from *X. laevis* oocytes in an environment that favours the native monomeric state. An alignment of the 23 Å representation revealed significant overlap with the surface model generated from the bacterial homologue, suggesting that the preliminary model may be sufficient for determining putative binding sites.

Within the oocyte system, hGLUT9 had good expression with sufficient protein yields to allow for gel filtration and isolation of the monomeric state. In the past, we have found that the detergent DDM was suitable for structural analysis of membrane proteins isolated from oocytes and verified this process indeed worked well for hGLUT9. Over expressed protein was observable in as little as ¼ of an oocyte and the subsequent isolated protein was detected in Western blot and silver stain. Protein was further purified by size-exclusion chromatography. Fraction 35 was chosen from this gel filtration step as corresponding to the highest percentage of monomeric hGLUT9 (**Figure S2**). Isolation of a single purified oligomeric state improves subsequent single particle reconstruction, however we demonstrate that multiple states do exist. The protein is likely represented in significant quantities as a dimeric or tetrameric assembly and due to the DDM detergent and purification steps, the monomeric form predominates. While Fraction 35 of the SEC is monomerically enriched, **Figure S2** demonstrates the presence of the multimeric form of hGLUT9. Evidence for tetrameric assembly of other GLUT family members has been demonstrated previously, but this is the first report of the multimeric potential of human GLUT9 [21]. An interesting follow up experiment would be to confirm the native oligomeric state stabilized through cross-linking experiments which would be more suitable for single particle reconstruction because of its size and supramolecular organization.

The *X*. *laevis* oocyte expression system offers the ability to simultaneously express human membrane protein and functionally verify the expression through biochemical and electrophysiological experimentation. With the use of the robotized HiClamp two-electrode voltage clamp system, we were able to rapidly verify functional expression of GLUT9. Oocytes expressing hGLUT9 demonstrate strong phloretin sensitive signals averaging 80 nA. Phloretin, a non-specific inhibitor of hGLUT9, reduced the current by nearly 50%, which is consistent with previous work [3]. The surface expression of hGLUT9 was verified by two independent experiments. Like all members of the GLUT family, hGLUT9 contains an extracellular glycosylation site. This post-translational modification attaches a carbohydrate through an enzymatic reaction within the ER subsequent to surface expression of hGLUT9. Protein deglyocosylation via application of PNGase revealed a significant shift in the mobility of hGLUT9 suggesting that the majority of expressed protein was glycosylated. While deglycosylation indicates the proper expression and folding of hGLUT9, the potential remains that the glycosylated protein may still not be expressed at the cell surface. To further indicate the presence of hGLUT9 at the plasma membrane we performed membrane biotinylation, in which surface labelled protein was isolated for hGLUT9 expression indicating the majority of expressed hGLUT9 was indeed present at the plasma membrane. Through the combination of electrophysiology, enzymatic deglycosylation, and surface biotinylation, it was revealed that the oocyte expression of hGLUT9 produced significant amounts of properly folded, functional protein at the plasma membrane. For future structure-function analysis, the oocyte system remains a solid model for the functional expression and purification of human hGLUT9 protein.

From 1500 injected oocytes, the amount of purified protein was sufficient to establish gel filtration chromatography and isolate different states of the protein. Monomeric form was separated from aggregate in the objective to obtain pure and homogenous sample for subsequent structural investigation by TEM in negative staining. The quantity of protein isolated, approx. 15 µg/ml, permitted single particle analysis and 3D reconstruction. The class averages allowed for the generation of an initial 3D model with sufficient similarity to our predictive crystal structure allowing for additional refinement to reach a 23 Å resolution. The final density map overlays well with the homology-based model but contains a few regions that extend beyond the predicted model. The differences arise from the origins of the XylE structure used to determine our original model. When deriving a crystal structure, the crystal lattice is constrained to one conformation whereas SPR involves numerous conformations and diversity, especially among regions which are dynamic and unstructured, as is likely the case for the C-terminal domain and extracellular loop marked by an asterisk (*) in **Figure 7**. The differences in our observations obtained from SPR and homology-based modelling could reflect the conditions used in our approach where the protein is solubilized, physically absorbed to a grid, and finally stained. Moreover, hGLUT9 is considered too small to expect fine details in the structure due to the resolution restrictions when using negative stain techniques. The contrast observed is due to the staining, or protein “shadow”, and not to the physical structure of the protein, as would be the case in cryo-EM. Cryo-EM can be significantly more sensitive with 3D reconstruction possible at medium resolution [22]. However, the number of particles required is exponentially higher due to the lower levels of contrast and decreased signal to noise ratios observed in cryo-EM. Moreover, for all TEM methods, detergents surround the solubilized membrane protein. These detergents become part of the overall particle and could contribute to the conformation of the protein or permit the interpretation of a false extension or form within the structure. This is made more evident using negative staining, as detergents appear on the micrograph as high-contrast signals similar to protein. Nevertheless, our result indicate our predictive model is defined well within the density map and serves to provide initial evidence towards our goal to determine if the predicted model is suitable for docking approaches that will help to identify the amino acids involved in the binding and transport of urate.

**Figure 8:**
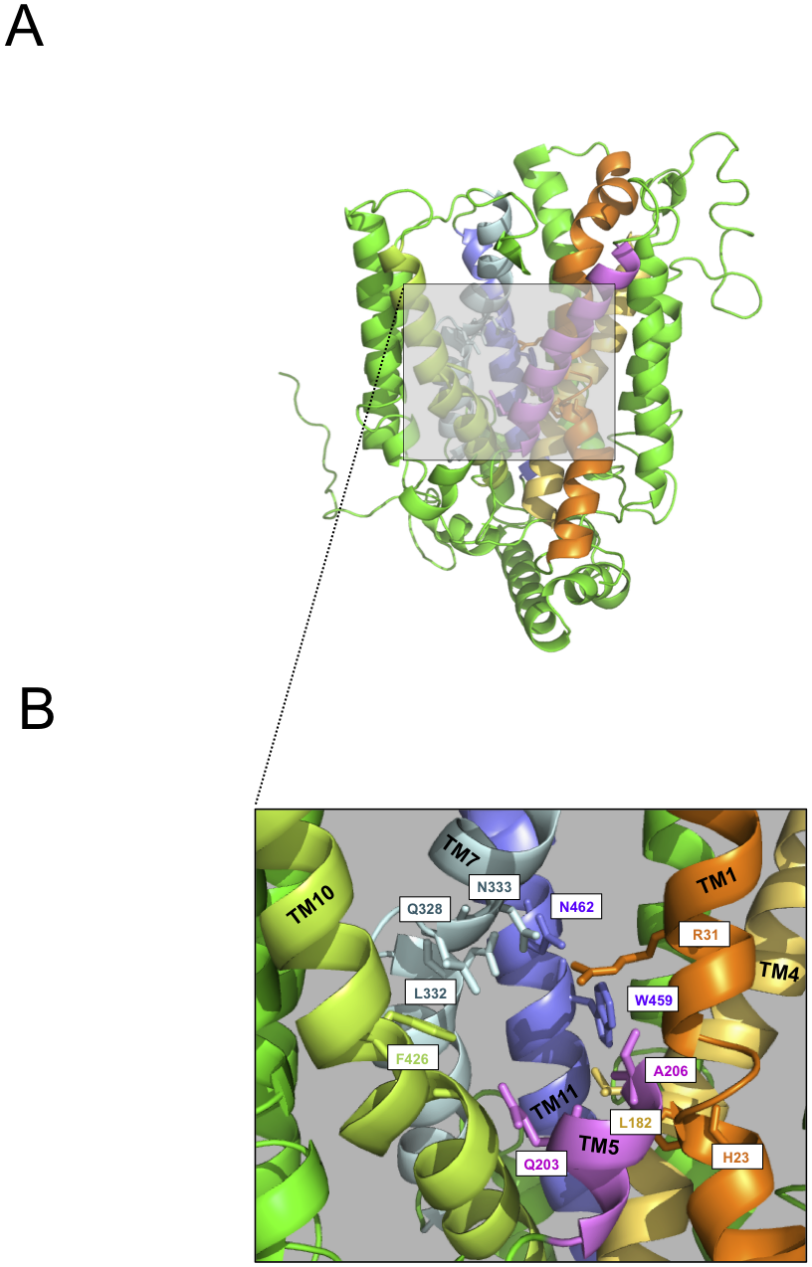
Hypothetical Substrate Binding Pocket in hGLUT9 model. (A) The surface modeling of hGLTU9, as described in Figure 2, was used to determine a 3 Å- pocket that could serve as a hypothetical substrate-binding site. (B) The putative binding site found is formed by amino acids: H23, R31, L182, Q203, A206, Q328, L332, N333, F426, W459 and N462.

As an exercise to determine the potential benefits of the homology based hGLUT9 structure, we created a surface model using PyMOL v0.99. Here we discovered an interior cavity aligned to the binding pocket for D-xylose co-crystallized with the bacterial homologue XylE. While subsequent work will need to validate our hyopthesis, it could be extrapolated that this is the binding pocket for urate in hGLUT9. **Figure 8** describes this cavity in which we extrapolated from the surface model the amino acids potentially involved in the binding of the urate: H23, R31, L182, Q203, A206, Q328, L332, N333, F426, W459 and N462. The putative urate binding site domain is constrained within trans-membrane region 1 (TM1-orange), TM4 (yellow), TM5 (purple), TM7 (cyan), TM10 green, and TM11 (blue). These results generate a restricted list of potential sites for subsequent mutagenesis. The next step will utilize the predictive structure of hGLUT9 to define docking simulations with urate and verify the precise amino acids interacting with the substrate. Structure function relationship will be planed with single or combined mutagenesis and the function will be accessed by previously defined electrophysiology combined with radiolabelled C14 urate uptake assays. The identification of the urate binding site will be compared to the glucose-binding site of hGLUT1–4 in order to reveal the mystery of substrate specificity in hGLUT9 and potentially lead to novel pre-clinical modulators for this physiologically relevant transporter.

In general, our results satisfied our goals to express and purify functional human GLUT9 in the *Xenopus laevis* model system. We went an additional step towards subsequent structure-function studies by creating a homology-based structure of hGLUT9. We verified the overall strategy by aligning our model within a 23 Å single particle reconstruction of the purified human protein. A putative binding pocket for the substrate urate was determined based off of the homology model, leaving the door open for site-directed mutagenesis and a greater understanding of the biophysical properties of one of the most distinct and physiological significant members of the SLC2 family members.

## Supporting Information

Figure S1 **Sequence Alignment for XylE and SLC2A human family homologues.** Similar color code for amino acids was chosen as Figure 1. Alignment was performed using SeaViewer 4 NCBI Blast sequences.
(TIFF)

Figure S2 **Single particle analysis of Fraction 35 reveals potential multimeric forms of hGLUT9.** (A) Representative electron micrograph of negative stained monomeric and dimeric hGLUT9 particles resulting from fraction 35 of the size exclusion chromatography (SEC). Black rings represent the dominant monomeric form, while white rings indicate possible dimeric particles as demonstrated by the SEC analysis below. Scale bar is 75 nm. (B) Individual images representing monomeric and dimeric particles isolated from the micrograph. Scale bar is 13 nm.
(TIFF)
