# Supplementary figures and images for: Expression, Purification, and Structural Insights for the Human Uric Acid Transporter, GLUT9, Using the Xenopus laevis Oocytes System
Source: PLoS One. 2014 Oct 6;9(10):e108852. doi: 10.1371/journal.pone.0108852 (PMC4186817; doi:10.1371/journal.pone.0108852)

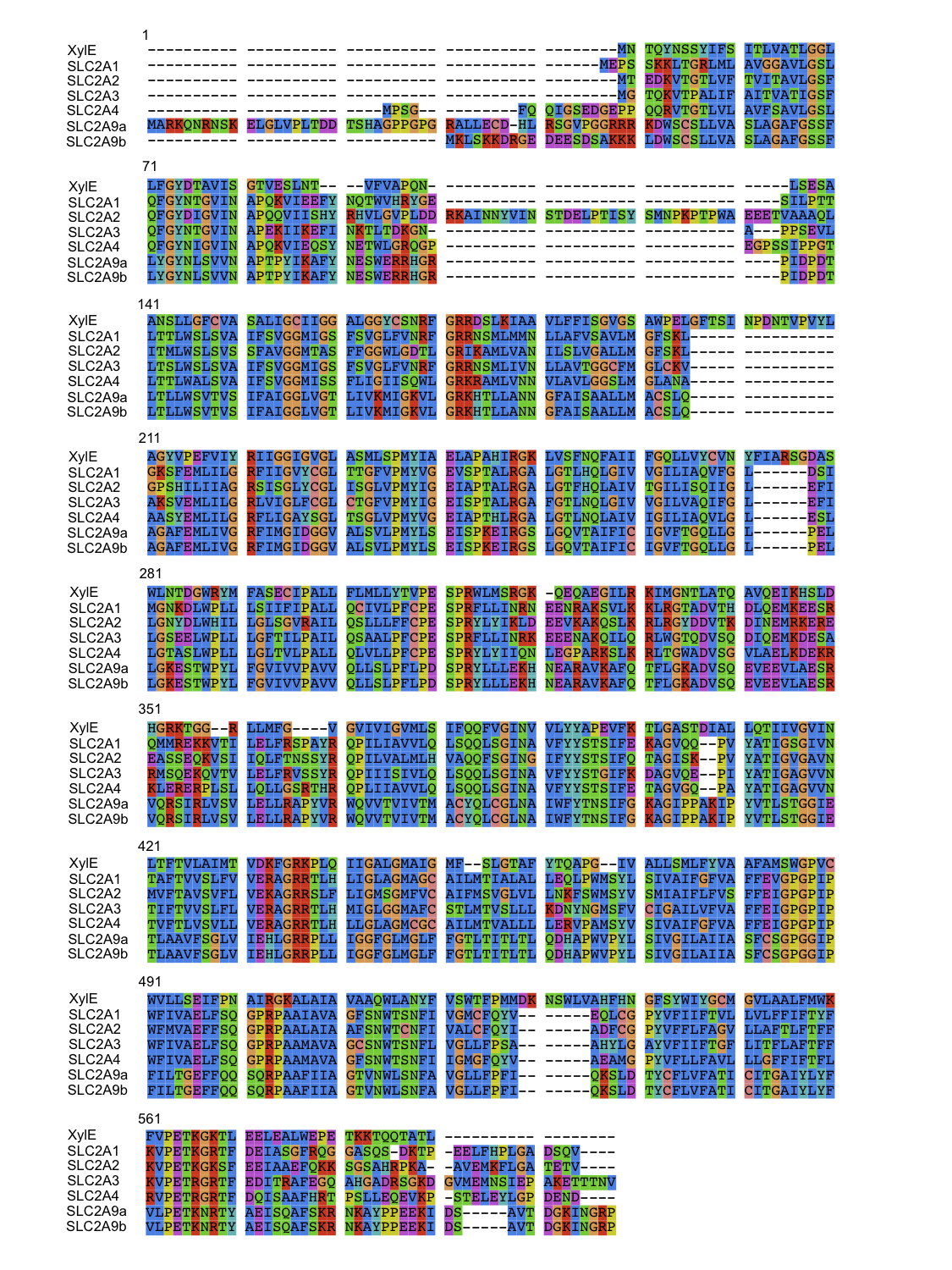

Supplement: Figure S1 — Sequence Alignment for XylE and SLC2A human family homologues. Similar color code for amino acids was chosen as Figure 1. Alignment was performed using SeaViewer 4 NCBI Blast sequences. (TIFF) [file pone.0108852.s001.tiff]

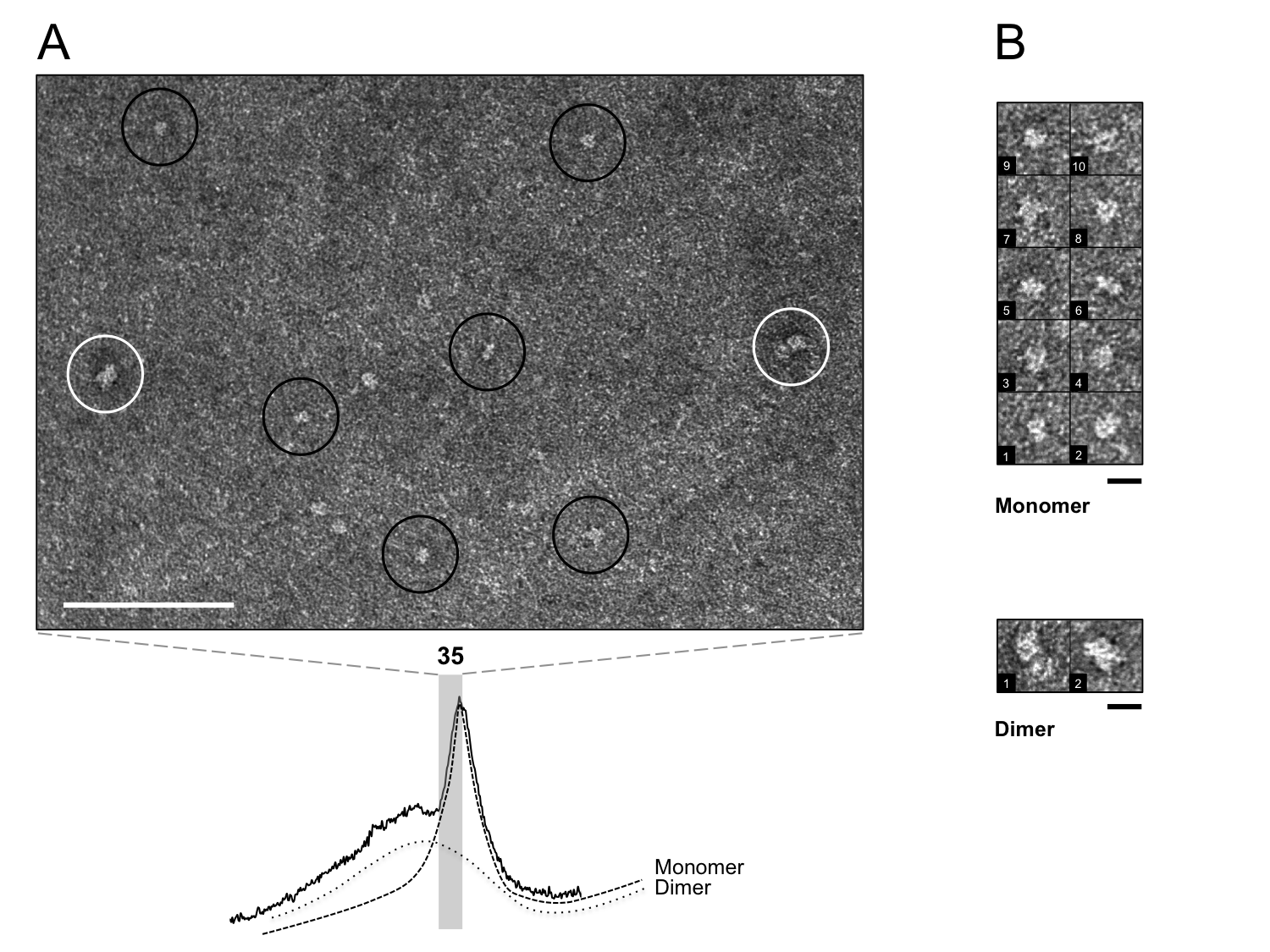

Supplement: Figure S2 — Single particle analysis of Fraction 35 reveals potential multimeric forms of hGLUT9. (A) Representative electron micrograph of negative stained monomeric and dimeric hGLUT9 particles resulting from fraction 35 of the size exclusion chromatography (SEC). Black rings represent the dominant monomeric form, while white rings indicate possible dimeric particles as demonstrated by the SEC analysis below. Scale bar is 75 nm. (B) Individual images representing monomeric and dimeric particles isolated from the micrograph. Scale bar is 13 nm. (TIFF) [file pone.0108852.s002.tiff]
